# Massive splenomegaly in hairy cell leukemia causing urinary retention

**DOI:** 10.1002/ccr3.3624

**Published:** 2020-12-04

**Authors:** Mohammad Ammad Ud Din, Joel Shapiro, Ronald Sham

**Affiliations:** ^1^ Department of Internal Medicine Rochester General Hospital Rochester NY USA; ^2^ Department of Pathology and Laboratory Medicine Rochester General Hospital Rochester NY USA; ^3^ Department of Hematology & Oncology Rochester General Hospital Rochester NY USA

**Keywords:** hairy cell leukemia, hematology, lymphoma

## Abstract

Hairy cell leukemia can cause massive splenomegaly which may lead to urinary retention and constipation. Patients usually require a splenectomy for relief of symptoms.

## Graphical abstract

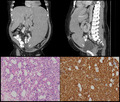

A 56‐year‐old male with hairy cell leukemia (HCL) presented with urinary retention. A computed tomography (CT) scan of the abdomen revealed massive splenomegaly with a mass effect on the bladder. His symptoms were relieved after splenectomy was performed. He was later started on vemurafenib.

A 56‐year‐old gentleman with hairy cell leukemia (HCL) presented with abdominal discomfort, constipation, and urinary retention. The laboratory tests showed hematocrit of 24%, white blood cell count of 10 600/uL, and platelet count of 25 000/uL. A computed tomography (CT) scan of the abdomen revealed massive splenomegaly measuring 35 cm craniocaudally with a mass effect on the bladder (Figure 1). He had been diagnosed with hairy cell leukemia 7 years ago when he presented with fatigue and pancytopenia. A CT‐guided bone marrow biopsy was performed to confirm the diagnosis (Figure 1). After failing cladribine, he was treated with rituximab but developed a severe reaction and had to be switched to pentostatin. However, a complete response could not be achieved.

**FIGURE 1:**
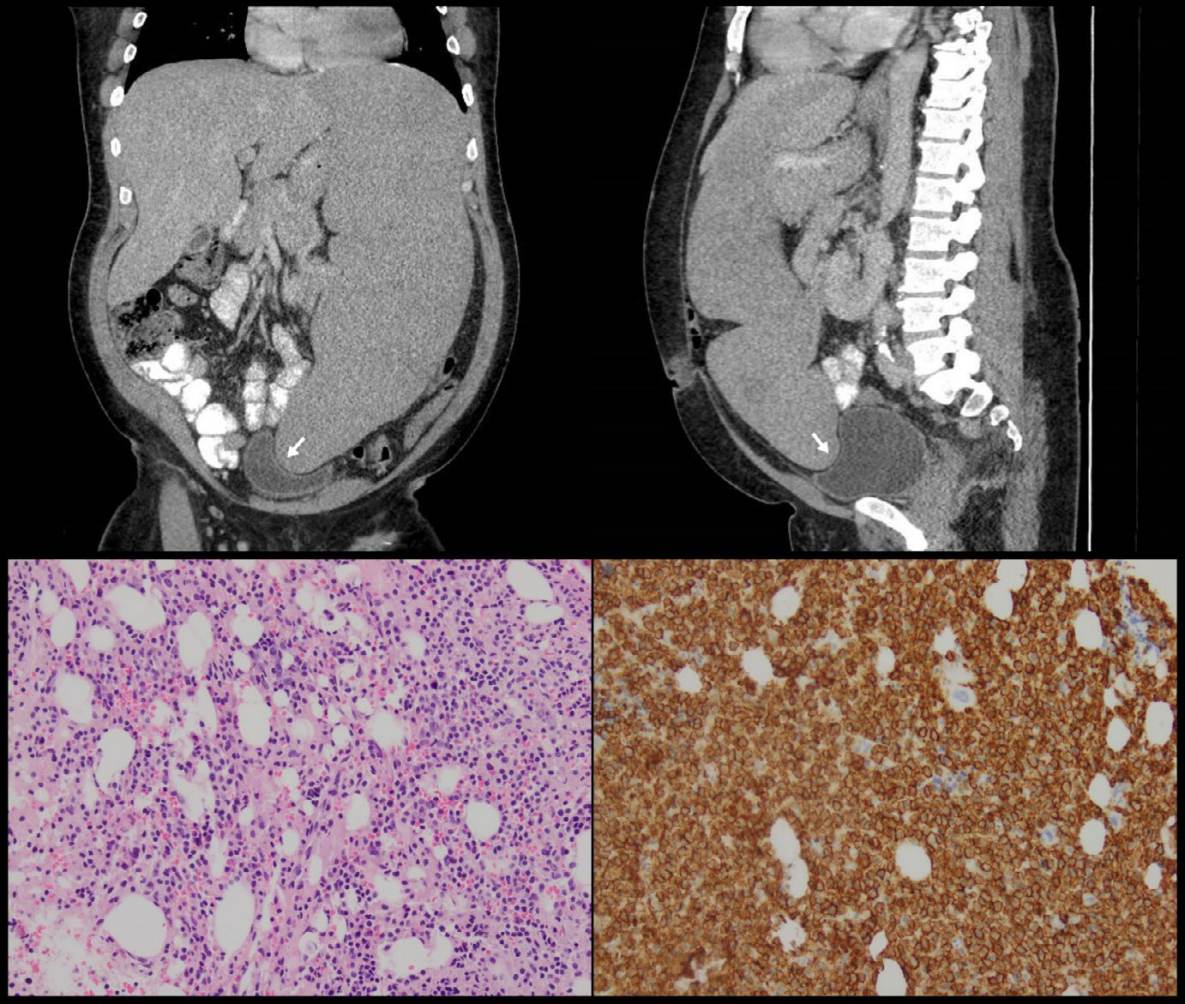
Coronal (upper left) and sagittal (upper right) views of a noncontrast computed tomography (CT) scan of the abdomen/pelvis showing massive splenomegaly. The spleen can be seen extending into the pelvis with a mass effect on the bladder (white arrows). Hematoxylin and eosin staining of the bone marrow showing the characteristic infiltration by atypical lymphocytes with abundant eosinophilic cytoplasm and irregular margins (lower left). Immunohistochemical staining reveals the atypical lymphocytes to stain strongly positive for CD 20, confirming their origin as B cells (lower right)

The mean age of diagnosis of HCL is around 65 years, and the hallmark finding on physical examination is splenomegaly.^1^ The malignancy accounts for approximately 2% of all leukemias and derives its name from the characteristic atypical “hairy” B cells, which can be seen on the peripheral blood smear, trephine bone marrow biopsy, or aspirate.^2^ The patient underwent splenectomy, which resolved his symptoms. Four months later, he was started on vemurafenib as molecular studies revealed BRAF mutation‐positive malignant cells.

## CONFLICT OF INTEREST

The authors declared no potential conflicts of interest with respect to the research, authorship, and/or publication of this manuscript.

## AUTHOR CONTRIBUTIONS

MAUD: wrote the manuscript. JS: was the primary pathologist on the case and provided the images with description for the manuscript. RS: was the primary hematologist on the case and critically reviewed the manuscript and made final edits prior to the submission.

## ETHICAL APPROVAL

This report for a clinical image was conducted in accordance with the Declaration of Helsinki. The collection and evaluation of all protected patient health information was performed in a Health Insurance Portability and Accountability (HIPAA) complaint manner. A formal informed consent was obtained from the patient prior to the publication of this article.

## Data Availability

Data sharing not applicable to this article as no datasets were generated or analyzed during the current study.
